# Beyond the Gut: Necrotizing Enterocolitis as a Gut–Brain Axis Disorder of Neurodevelopment

**DOI:** 10.3390/biomedicines14040780

**Published:** 2026-03-30

**Authors:** Monica D. Jordan, Lalit Agarwal, Chhinder P. Sodhi

**Affiliations:** 1 Robert H. Smith School of Business, University of Maryland, College Park, MD 20742, USA; mjorda15@umd.edu (M.D.J.); lagarwa1@umd.edu (L.A.); 2 Division of Pediatric Surgery, Department of Surgery, Johns Hopkins University School of Medicine, Baltimore, MD 21205, USA

**Keywords:** necrotizing enterocolitis, preterm infants, Toll-like receptor 4, gut–brain axis, microbiome, dysbiosis, metabolic network, formula feeding, intestinal barrier, inflammation, neuroimmune, immune cells

## Abstract

Necrotizing enterocolitis (NEC) is a major cause of illness and death in preterm infants and is increasingly linked to long-term neurodevelopmental issues among survivors. Usually seen as a gastrointestinal disease, NEC is rarely viewed from a brain-centered perspective. In this Perspective, we suggest that NEC should be understood as a disorder of the gut–brain axis affecting neurodevelopment. We combine clinical and experimental evidence showing that intestinal inflammation, microbial imbalance, epithelial barrier failure, and systemic immune activation during NEC all contribute to the disruption of early brain development. We contend that neurodevelopmental damage is a key feature of NEC rather than just a secondary effect of prematurity. Recognizing NEC as a gut–brain axis disorder is crucial for research models, treatment approaches, and assessing long-term outcomes in affected infants.

## 1. Introduction

Necrotizing enterocolitis (NEC) is a devastating disease in preterm infants, involving intestinal inflammation, barrier failure, and high morbidity and mortality [1,2]. Advances in neonatal care have improved survival from NEC, but long-term neurodevelopmental disabilities like cognitive delay, executive dysfunction, learning difficulties, and behavioral problems have become more apparent in the NEC survivors [3,4,5]. Rather than being inherent to NEC itself, these neurodevelopmental deficits are often attributed to prematurity or critical illness. The focus in clinical and basic research settings remains on NEC-induced intestinal injury, sepsis, and survival, while neurodevelopmental outcomes are studied without a mechanistic understanding. This gap also exists in the gut–brain axis literature, where early intestinal inflammatory diseases are seldom viewed as causal factors of neurodevelopmental injury despite biological plausibility. The gut–brain axis involves bidirectional communication through neural pathways (including gut enteric neurons and enteric glia beside central and peripheral neurons), immune pathways, enteroendocrine pathways, and microbial pathways, which are actively developing during early life [6,7]. Disruption of gut–brain communication during critical developmental periods—such as that caused by NEC, which leads to severe inflammation, microbial imbalance, and systemic immune activation, resulting in neurological injury—may have long-term effects [3,6,8]. Mechanistically, neurodevelopmental injury is often linked to factors such as prematurity, hypoxia, or medication exposure, but these do not fully explain the connection between NEC severity and cognitive outcomes or the evidence linking intestinal inflammation to brain injury. The developing brain’s susceptibility to inflammatory signals is increased in preterm infants, affecting microglia, myelination, and synaptic pruning, all of which are essential for cognition [9,10]. NEC-associated inflammatory surges and microbiome dysbiosis—characterized by decreased diversity and pro-inflammatory taxa—may directly impair neurodevelopment, creating a feedback loop [11,12]. The enteric nervous system (ENS) is also affected by NEC, disrupting CNS-gut balance, with microbiome changes contributing to ENS dysfunction [2,6]. The colon, which produces over 90% of serotonin and Short-chain fatty acids (SCFAs), plays a vital role in gut–brain health and should be studied earlier in research [7]. Recognizing NEC as a gut–brain axis disorder and understanding these interconnected mechanisms are essential for developing targeted neuroprotective strategies. This perspective argues that NEC should be regarded as a major and biologically active contributor to neurodevelopmental injury, acting through gut–brain axis mechanisms during critical developmental windows. While prematurity, hypoxia, and intensive care exposures remain important contributors, accumulating experimental and clinical evidence indicates that NEC itself exerts independent and mechanistically distinct effects on the developing brain.

## 2. Mechanistic Pathways Linking NEC to Neurodevelopmental Injury

NEC instigates neurodevelopmental injury through the gut–brain axis, involving interconnected processes such as intestinal inflammation, dysregulated enteric nervous and endocrine functions, microbial imbalance, and barrier failure. These NEC-induced disruptions increase neurological vulnerability during critical brain development, and understanding this cascade is essential for developing targeted interventions.

### 2.1. Intestinal Inflammation as a Primary Neurodevelopmental Insult

NEC involves severe intestinal inflammation caused by exaggerated Toll-like receptor 4 (TLR4)-mediated innate immune activation in the immature gut, leading to intestinal epithelial injury, apoptosis, necroptosis, necrosis, intestinal barrier dysfunction, and the translocation of pro-inflammatory cytokines and chemokines into systemic circulation, crossing the immature blood–brain barrier [2,9]. In preterm infants, this response is uncontrolled, characterized by increased pro-inflammatory cytokines and prolonged immune activation, coinciding with critical phases of brain development [3,9]. Preclinical studies demonstrate that excessive TLR4-driven inflammation during critical windows of oligodendrocyte progenitor proliferation can impair myelination and synaptic pruning [9,10]. Therefore, intestinal inflammation in NEC acts as a neurodevelopmental stressor with long-term effects.

### 2.2. Microbiome Dysregulation and Loss of Neuroprotective Signaling

NEC is increasingly recognized as preceded and accompanied by decreased microbial diversity and an increase in taxa linked to pro-inflammatory signaling [13]. While microbial dysbiosis is often viewed as increasing the vulnerability of the intestine, it also has significant effects on brain development [11]. Microbial colonization of the developing intestine plays a crucial role in shaping immune tolerance, metabolic signaling, and neurodevelopmental pathways by producing bioactive metabolites such as short-chain fatty acids and modulating the production of Serotonin by the enterochromaffin cells and by influencing the host’s immune responses to pathological cues [7,12]. In NEC, dysbiosis is linked to a reduced availability of microbial signals that support immune regulation and neural balance [14]. The loss of these signals during a sensitive developmental window may exacerbate systemic inflammation and deprive the developing brain of key modulatory inputs. Importantly, microbiome disruption in NEC is not transient; prolonged antibiotic exposure and delayed microbial recovery can extend this period of altered signaling well beyond the acute phase of intestinal disease. This sustained dysregulation reinforces a feed-forward loop in which microbial imbalance and inflammation mutually amplify their effects on the gut–brain axis.

### 2.3. Barrier Failure Plays a Key Role in Gut–Brain Crosstalk

A key feature of NEC pathogenesis is the breakdown of the intestinal epithelial barrier, allowing microbial products and inflammatory mediators to enter the systemic circulation and further activate the immune system. Barrier failure worsens gut–brain disruptions by causing systemic endotoxemia and inflammation, activating immune cells, including endothelial cells, affecting microvasculature blood flow in the intestines and brain, and promoting neuroinflammation [2,9,15,16]. In preterm infants, weakened blood–brain barriers with increased permeability worsen this issue, allowing gut-derived inflammatory signals to trigger neuroinflammation and hinder brain development [9]. Thus, barrier dysfunction links intestinal problems to brain injury.

### 2.4. Integration of Mechanisms: A Feed-Forward Gut–Brain Injury Loop

Intestinal inflammation, microbiome imbalance, and barrier failure form an interconnected pathological cycle. Intestinal injury causes dysbiosis and barrier disruption, while dysbiosis worsens inflammation [17]. Barrier failure increases systemic exposure to gut mediators, and neuroinflammation impacts immune regulation and gut control. When this pathological cycle occurs during critical windows of brain development, neural circuit formation and cognitive potential may be durably altered. This challenges the idea that neurodevelopmental impairment in NEC is unavoidable due to prematurity. Instead, NEC triggers a modifiable gut–brain injury cascade. Recognizing this highlights the importance of evaluating interventions for gut integrity and neurodevelopmental risk, with implications for modeling, biomarkers, and therapies.

## 3. Neurocognitive Outcomes as a Core Phenotype of NEC

Long-term neurodevelopmental issues observed in NEC survivors support a gut–brain axis injury model. Both clinical and basic research associate NEC with cognitive delays, learning difficulties, and behavioral problems that persist long after NEC resolves or its outcome is determined. Although these issues are often blamed on prematurity or intensive care, the ongoing neurocognitive deficits suggest a direct biological link between intestinal disease and neurodevelopmental outcomes.

### 3.1. Cognitive Outcomes

Cognitive outcomes are among the most common neurodevelopmental issues in NEC survivors, who often score lower on assessments of intelligence, language, and executive function compared to preterm peers without NEC [3,4,11]. NEC-related inflammation and neuroimmune activation occur during critical stages of oligodendrocyte development and myelination, which are vital for neural signaling [9,10]. The degree of cognitive impairment correlates with the severity of NEC disease burden, clearly demonstrating a dose-dependent relationship between NEC severity and neural outcome rather than mere coincidence.

### 3.2. Behavioral and Learning Outcomes

NEC survivors often face cognitive, behavioral, and learning challenges, including attention difficulties, emotional regulation difficulties, and academic hurdles [4]. These issues can be subtle at first but tend to become more noticeable as children grow older and cognitive demands increase, reflecting early disruptions in neural circuits that support executive and social–emotional skills. From a gut–brain axis perspective, early NEC-induced [6,7] neuroinflammation primes microglia, potentially interfering with synaptic pruning and network formation and leading to lasting changes in brain circuits [3,9,10,11]. Moreover, a persistent imbalance in the gut microbiome after NEC could affect neurodevelopment through abnormal immune and metabolic signals, potentially extending the effects of injury into infancy [11].

### 3.3. Reframing Neurodevelopmental Outcomes in NEC

Neurocognitive phenotypes in NEC survivors challenge the idea that NEC-induced brain injury is unavoidable because of prematurity or critical illness. Instead, they support a model in which NEC pathology initiates a non-physiological/pathological gut–brain axis cascade that negatively affects neurodevelopment. The consistent cognitive, structural, and behavioral outcomes in NEC survivors suggest a shared biological basis rather than random injury. This shift has important implications for research and clinical practice. Neurodevelopmental outcomes should be viewed as part of NEC severity and incorporated into disease definitions, endpoints, and treatments. Any plausible intervention that only reduces intestinal NEC injury but does not address systemic or neuroinflammatory responses might be insufficient to protect brain function and long-term neurodevelopmental outcomes. Strategies that support gut health, restore microbiome balance, or reduce harmful immune responses during critical periods could benefit both intestinal and neurodevelopmental health. Explicitly linking gut-derived pathology to brain phenotype positions NEC as both a neonatal gastrointestinal emergency and a developmental brain disease mediated by the gut–brain axis. Recognizing this dual role is essential for advancing mechanistic understanding and facilitating translational progress in the care of infants with NEC.

## 4. Limitations of Current NEC Interventions

Standard NEC management relies on broad-spectrum antibiotics, bowel rest, and supportive care [18]. Although necessary in the acute setting, these interventions may unintentionally worsen gut–brain axis dysfunction. Early-life antibiotic exposure disrupts colonizing microbiome communities, delaying the recovery of microbial diversity and functional signaling essential for immune and neurodevelopmental maturation [19,20,21]. From a gut–brain perspective, such disruption may extend maladaptive inflammatory and metabolic signaling to the developing brain, compounding injury from the acute phase. Similarly, probiotic strategies, although promising for reducing NEC incidence [22], have often been implemented without mechanistic stratification or neurodevelopmental endpoints. Most clinical trials focus on NEC prevention rather than post-diagnosis neuroprotection, and few assess whether microbiome modulation alters systemic inflammation or long-term cognitive outcomes. This disconnect reveals a broader translational gap: interventions are evaluated solely for their effects on intestinal pathology, despite evidence that brain injury may occur in parallel or downstream. Importantly, the limited neurodevelopmental impact observed in previous microbiome- and anti-inflammatory-based interventions warrants careful consideration. Several probiotic and nutritional studies have demonstrated reductions in NEC incidence but have not consistently resulted in improved long-term neurodevelopmental outcomes. This discrepancy may arise from multiple factors, such as late intervention timing relative to critical neurodevelopmental periods, insufficient targeting of functional microbial signaling pathways, and the omission of neurodevelopmental endpoints in trial design. These findings do not disprove the gut–brain axis theory but instead highlight the need for mechanism-driven, time-sensitive interventions that specifically target neuroimmune programming rather than solely focusing on intestinal issues.

## 5. Nutrition as a Gut–Brain Therapeutic Modulator

Early-life nutrition is one of the most crucial and underutilized factors influencing the neonatal gut–brain axis [23]. Mothers’ own milk (human breast milk), in particular, offers not only optimal nutrition but also bioactive components like growth factors, rich in IgA, IgM, and IgG antibodies, neuropeptides, hormones, and anti-inflammatory agents that impact microbial colonization, enhance barrier integrity, and regulate immune responses [24,25]. These qualities position nutrition as a potential dual-action therapy capable of reducing both intestinal injury and neurodevelopmental risk [26]. When human breast milk is unavailable, non-human synthetic formula-based nutritional strategies typically focus on improving feeding tolerance and gut protection, while meeting high energy needs, but place limited emphasis on neurodevelopmental signaling. A gut–brain perspective highlights the importance of evaluating how nutritional interventions influence systemic inflammation, microbial metabolite production, and brain development. Timing is especially vital; nutritional interventions during or immediately following NEC may exert greater influence on long-term outcomes than those implemented later.

## 6. Brain-Informed NEC Therapeutics

A gut–brain axis perspective also opens new avenues for therapeutic innovation. Anti-inflammatory strategies that safely attenuate systemic immune activation during NEC could reduce downstream neuroinflammatory injury, provided they are carefully tailored to the immature immune system. Similarly, microbiome-targeted interventions designed to restore functional microbial signaling—rather than simply increasing taxonomic diversity—may offer neuroprotective benefits if applied during defined developmental windows [27]. Crucially, these approaches require a shift in how translational success is defined. NEC therapies should be evaluated for their capacity to preserve neurodevelopmental trajectories, not solely for their effects on survival or intestinal healing. This shift necessitates the incorporation of brain-relevant biomarkers, such as inflammatory profiles, microbial metabolites, and neuroimaging measures, into clinical trials and experimental models.

## 7. Integrating Gut–Brain Outcomes into Clinical Practice and Research

From a clinical standpoint, recognizing neurodevelopmental impairment as a core NEC outcome supports earlier and more systematic neurodevelopmental surveillance for affected infants. Risk stratification models that incorporate markers of gut–brain axis disruption could identify infants most likely to benefit from targeted interventions. In research settings, experimental models of NEC should be explicitly designed to assess brain outcomes alongside intestinal pathology, enabling more accurate evaluation of therapeutic efficacy. More broadly, these reframe the field to move beyond organ-specific silos. NEC management has traditionally been dominated by gastrointestinal and surgical considerations, while neurodevelopmental follow-up occurs downstream and often in isolation. A gut–brain axis framework argues for integrated care pathways that align neonatal, nutritional, immunological, and neurodevelopmental expertise from the outset.

## 8. Future Directions and Call to Action: Advancing a Neurodevelopment-Centered NEC Paradigm

Reframing NEC as a gut–brain axis disorder carries important implications for the future trajectory of both research and clinical care. While the biological plausibility linking intestinal inflammation, microbial dysregulation, and neurodevelopmental injury is increasingly clear, meaningful progress will require deliberate shifts in study design, outcome prioritization, and therapeutic evaluation. The field now faces an opportunity—and an obligation—to move beyond survival-centered metrics toward an integrated framework that explicitly accounts for brain development as a primary outcome of NEC.

### 8.1. Redefining Experimental and Clinical Endpoints

A key initial step in studying neurodevelopmental outcomes in NEC survivors is to include systemic circulation in the gut–brain axis. Experimental models should be designed to evaluate brain structure, neuroinflammatory signaling, and functional outcomes, as well as intestinal and systemic health, rather than treating the brain as a secondary or optional endpoint. Likewise, clinical studies and trials need to go beyond short-term survival and gastrointestinal recovery to incorporate standardized, long-term assessments of cognition, behavior, and executive function. Importantly, neurodevelopmental endpoints should be assessed at multiple stages, not just in late childhood, to detect evolving phenotypes that may only emerge as cognitive demands increase. Redefining NEC severity to encompass neurodevelopmental risk would be a major advancement in understanding and assessing disease burden.

### 8.2. Building Integrated Gut–Brain Cohorts

Advancing our understanding of NEC-related brain injury will rely on long-term, multidimensional studies that combine data from intestinal health, microbiota, immune responses, and neurodevelopment. These studies should track the evolving relationship between gut issues and brain development over time instead of using only static or cross-sectional snapshots. Combining microbiome analysis, systemic inflammation markers, metabolic profiles, and neuroimaging will be crucial to uncover mechanisms and find predictive biomarkers. Such integrated research will also help categorize risks, identifying infants more likely to suffer gut–brain axis-related damage. Stratified methods are vital because of the varied ways NEC presents and its outcomes, and they may clarify why some survivors face severe neurodevelopmental challenges while others do not.

### 8.3. Timing as a Central Factor in Intervention Success

Future NEC treatment strategies must account for the timing of neurodevelopment. The link between NEC and neurological injury occurs during early, critical periods of brain development, when gut-derived inflammatory and microbial signals can cause significantly more harm. Treatment strategies that only prevent intestinal necrosis might not protect the brain if administered too late to influence neuroimmune programming. The timing of NEC treatment is essential for designing trials and making clinical decisions. Essentially, NEC therapies targeting inflammation, the microbiome, or barrier integrity should be evaluated for both effectiveness and timing relative to key stages of brain development. Recognizing issues in the gut–brain axis early could enable interventions that prevent long-term brain injury, even after the initial intestinal disease has resolved.

### 8.4. Mechanism-Based Therapies

A gut–brain axis framework requires shifting from simple empirical approaches to broad mechanism-based therapeutics. Instead of focusing solely on taxonomic alterations in the microbiome or on broad-spectrum inflammation suppression, future strategies should aim to re-establish functional signaling pathways that promote homeostasis and support neurodevelopment in premature neonates who survive NEC episodes. This involves identifying microbial metabolites, immune mediators, and host responses that serve as central links between gut pathology and brain outcomes. Equally important is assessing the unintended neurodevelopmental effects of current therapies. Antibiotics, nutritional modifications, and surgical interventions may differentially influence gut–brain signaling, and these effects should be examined thoroughly rather than assumed harmless. Incorporating neurodevelopmental considerations into therapeutic evaluation will help ensure that improvements in survival are not offset by preventable long-term morbidity.

### 8.5. A Call to Action for the Field

The collective evidence indicates a paradigm shift in the study and management of NEC and in the long-term outcomes of NEC survivors, especially neurodevelopmental outcomes. The gut–brain axis provides a comprehensive framework that integrates NEC-induced intestinal disease, systemic inflammation, microbial ecology, and brain development within a single biological system. Implementing this system-wide approach requires breaking down disciplinary boundaries and fostering collaboration among neonatology, neuroscience, immunology, and microbiome research. A neurodevelopment-focused approach to NEC is justified because it considers brain injury a fundamental aspect of the disease biology rather than merely a secondary effect. Aligning experimental models, clinical trials, and treatment strategies with the understanding of early-life gut–brain dynamics could enable interventions that improve survival rates and cognitive development. Additionally, NEC could serve as a model to understand the impact of early-life inflammatory diseases on long-term brain health, with implications extending beyond the neonatal period. Our research program applies this framework through integrated experimental and clinical approaches. These include (i) preclinical NEC models that assess intestinal injury, systemic inflammation, and region-specific brain effects; (ii) long-term clinical studies that incorporate microbiome profiling, circulating inflammatory markers, and neurodevelopmental assessments; and (iii) testing gut-targeted interventions, such as human milk-derived bioactives, for their potential to influence both intestinal and neurodevelopmental outcomes. These strategies aim to translate the gut–brain axis concept into practical, measurable endpoints.

## Data Availability

No data were used for the research described in the article.
